# Non-Communicable Diseases among Women of Reproductive Age Visiting the Department of Obstetrics and Gynecology of a Tertiary Care Hospital

**DOI:** 10.31729/jnma.8471

**Published:** 2024-02-29

**Authors:** Indra Yadav, Sabita Jyoti, Chunauti Bahik, Jiya Acharya, Anjana Bohaju, Siddhartha Kumar Yadav

**Affiliations:** 1 Department of Obstetrics and Gynaecology, Birat Medical College and Teaching Hospital, Biratnagar, Morang, Nepal; 2 Department of Community Medicine, Nepalgunj Medical College and Teaching Hospital, Nepalgunj, Banke, Nepal; 3 Kathmandu Medical College and Teaching Hospital, Sinamangal, Kathmandu, Nepal

**Keywords:** *chronic disease*, *non-communicable disease*, *prevalence*, *risk factors*

## Abstract

**Introduction:**

Non-communicable diseases are a significant cause of mortality worldwide, posing a substantial risk to women's health, as stated by the World Health Organization. In Nepal, a survey revealed that 10.5% of the population suffers from hypertension. The primary objective of this study was to determine the prevalence of non-communicable diseases among women of reproductive age visiting the Department of Obstetrics and Gynecology of a tertiary care hospital.

**Methods:**

A descriptive cross-sectional study was conducted at the Department of Obstetrics and Gynecology among women of reproductive age presented from 6 November 2023 to 6 January 2024. The data was retrieved from the medical record during 1 November 2023 to 1 December 2023. Ethical approval was taken from the Institutional Review Committee. Convenience sampling method was used. The point estimate was calculated at a 95% Confidence Interval.

**Results:**

The prevalence of non-communicable diseases was 608 (39.02%) (36.60-41.45, Confidence Interval). The mean age was 29.26±3.46 years. The most common non-communicable disease reported was hypertension 204 (33.55%) followed by chronic respiratory diseases 200 (32.89%) and diabetes mellitus 154 (25.34%).

**Conclusions:**

The prevalence of non-communicable diseases among women of reproductive age group was higher as compared to other studies done in similar setting. The study underscores the urgency for stakeholders to implement health education, early detection, and preventive strategies, emphasizing the necessity of targeted interventions and broader public health initiatives to address non-communicable diseases.

## INTRODUCTION

Non-communicable diseases (NCDs) are a significant cause of mortality worldwide.Common risk factors include low fruit/vegetable consumption, physical inactivity, smoking, and excessive alcohol consumption.^1^ The World Health Organization (WHO) has identified NCDs as a major threat to women's health, particularly in low- and middle-income countries, where they often affect women during their most productive years.^2^ NCDs can also have a significant impact on maternal health and pregnancy outcomes, with cardiovascular diseases (CVD), metabolic diseases, haematological diseases, mental illness, and neoplasms being the most prevalent during pregnancy.^3,4^

In India, chronic hypertension is particularly common among women, affecting approximately 30.8% of the population.^5^ Similarly, a study in Nepal found a hypertension prevalence of 10.5%, with current tobacco use being identified as a risk factor.^1^ However, there is limited data on NCD prevalence among women of reproductive age in Nepal.

The aim of this study was to find the prevalence of NCDs among the women of reproductive age visiting to the Department of Obstetrics and Gynecology of a tertiary care hospital.

## METHODS

A descriptive cross-sectional study was conducted among women who visited the Department of Obstetrics and Gynecology, Birat Medical College and Teaching Hospital, Biratnagar, Morang, Nepal. The data were taken from the hospital record from 6 November 2023 to 6 January 2024. The study duration was from 1 November 2023 to 1 December 2023. Ethical approval was taken from the Institutional Review Committee of Nepalgunj Medical College Teaching Hospital (Reference number: 23/080/081). The data of the women in the reproductive age group 18-49 years were included in the study. Those women with incomplete data were excluded from the study. The convenience sampling method was used. The sample size was calculated using the following formula:


$$\begin{array}{ll} \text{n} & ={\text{Z}}^{2}\times \frac{\text{p}\times \text{q}}{{\text{e}}^{\text{2}}}\\ & =1.96^{2}\times \frac{0.05\times 0.05}{0.03^{2}}\\ & =1,068 \end{array}$$

Where,

n = minimum required sample size
Z = 1.96 at a 95% Confidence Interval (CI)
p = prevalence taken as 50% for maximum sample size calculation
q = 1-p
e = margin of error, 3%

The calculated sample size was 1,068. However, 1,558 women were taken in the study.

The NCDs included were cardiovascular disorders, chronic hypertension, endocrine disorders including pre-existing diabetes mellitus, neurological disorders, psychiatric disorders, chronic kidney disease, chronic liver disease, chronic respiratory diseases, autoimmune disorders, and cancer. Other NCDs were collectively studied as a miscellaneous group.^6^

The collected data were entered and analyzed using Microsoft Excel 2017. The point estimate was calculated at a 95% CI.

## RESULTS

Out of 1,558 women of reproductive age, the prevalence of NCDs was 608 (39.02%) (36.60-41.45, 95% CI). Among them, 195 (32.07%) were pregnant and 413 (67.93%) were nonpregnant. The mean age was 29.26±3.46 years. The majority 205 (33.72%) of women were not doing any work except household work while 245 (40.29%) were self-employed and 158 (25.99%) were working.

**Table 1:** Demographic distribution of all reproductive-age women with non-communicable diseases (n = 608).

Age (years)	n (%)
18-29	304 (50)
30-39	212 (34.87)
40-49	92 (15.13)

Among pregnant women, primigravidawere 65 (33.33%)(Table 2).

**Table 2:** Characteristics of pregnant women with non-communicable disease (n = 195).

Characteristics	n (%)
**Gravida**	
Primigravida	65 (33.33)
Multigravida	112 (57.44)
Grand multipara	18 (9.23)
**Period of gestation (weeks)**	
<37	66 (33.85)
37-40	101 (51.79)
>40	28 (14.36)
**Type of pregnancy**	
Singleton	177 (90.77)
Multifetal	18 (9.23)

Hypertension was a common presentation seen in 224 (36.84%) followed by chronic respiratory disease in 200 (32.89%) (Table 3).

**Table 3:** Categories of NCD among women of reproductive age group (n = 608).

Disease	Total n (%)	Non pregnant women (n = 413)	Pregnant women (n = 195)
Hypertension	224 (36.84)	115 (27.84)	109 (55.89)
Chronic respiratory disease	200 (32.89)	34 (8.23)	166 (85.12)
Diabetes mellitus	154 (25.33)	50 (12.10)	104 (53.33)
Cardiovascular disease	38 (6.25)	10 (2.42)	28 (14.35)
Cancer	12 (1.97)	12 (2.90)	-
Anemia	176 (28.95)	38 (9.20)	138 (70.76)

Among the risk factors, the majority 273 (44.90%) of women reported eating an unhealthy diet followed by 200 (32.89%) physically inactive. More than 176 (28.94%) of women had two or more than two risk factors present (Table 4).

**Table 4:** Risk factors present among reproductive-age women (n = 608).

Risk factors	Total n (%)	Non pregnant women (n = 413)	Pregnant women (n = 195)
Unhealthy diet	273 (44.90)	79 (19.12)	194 (99.48)
Smoking or use of another form of tobacco products	64 (10.53)	60 (14.52)	4 (2.05)
Alcohol Consumption	41 (6.74)	29 (7.02)	12 (6.15)
Obesity/overweight	158 (25.98)	68 (16.46)	90 (46.15)

## DISCUSSION

The prevalence of NCDs among women of reproductive age was 39.02% in this study, which is notably lower than the 77% reported by the National Family Health Survey in India.^6^ However, the prevalence of NCD risk factors, as per the Bangladesh Demographic and Health Survey data from 20172018, was 34.55%.^6^ According to the NCD Alliance, two out of every three women die due to NCDs.^7^

The mean age of women in this study was 29.26±3.46 years, which is consistent with similar studies.^6,7^ Hypertension was prevalent among 224 women (40.13%) in this study, which is notably higher than the 10.5% reported in the 2016 Nepal Demographic Health Survey (NDHS). This variance could be attributed to the hospital-based nature of our study, which encompassed a diverse range of women, including those with predispositions to various comorbidities such as pregnancy-induced hypertension. These additional factors might have contributed to the higher prevalence observed.^1^

Similarly, the prevalence of hypertension was 4.51%, 20.3%, and 5.5% among women in the National Family Health Survey India, Bangladesh, and Africa respectively.^6-8^ In this study the prevalence of hypertension was 36.84%. Among pregnant women, the proportion of hypertension is 48.66%, chronic respiratory disease in 83%, and diabetes mellitus in 67.53%.^5^

The proportion of anaemia in this population was 28.95%. Other studies have stated as much as 57.2% of the similar population suffered from anaemia.^6^ Diabetes mellitus was observed in 25.34%. Other studies showed the prevalence of diabetes to be 8.24% and heart disease to be 0.74%.^6^

Among the risk factors, the majority 44.90% of women reported eating an unhealthy diet and being physically inactive 32.89%. In this study, 64 individuals (10.53%) were observed to smoke or use other forms of tobacco products. This prevalence is slightly higher than the 8.9% reported in the 2016 NDHS, a nationally representative survey conducted periodically.^1^ There are still 2.05% of pregnant women with tobacco use which deserves attention because of its adverse maternal and child health during the perinatal period and long-term harms. Similarly, the nationwide survey of Bangladesh showed a prevalence of smoking to be 9.6%.^7^ That in Africa was 2.4%.^8^

Similarly obesity/overweight was observed in 25.98% which was a little bit higher than the 22.2% observed in NDHS data. Additionally, the study revealed that overweight/obesity rates were particularly elevated among elderly individuals, married women, and those in the wealthiest quantile.^1^ The survey of India and Bangladesh showed that to be 27.15% and 31.6% respectively.^6,7^ Similarly, surveys from sub-Saharan African countries showed the prevalence of overweight/obesity to be 6.7-44.5% with an average of 23.1%.^8^ Alcohol Consumption in this population was observed in 6.74%. Sub-Saharan African countries showed that prevalence of 23.9%.^8^

This study has several limitations. Firstly, it was conducted at a single hospital, which may not be representative of the entire population of women in the reproductive age group in Nepal. Additionally, the use of convenience sampling may have introduced bias by selecting participants who were more readily available or accessible, potentially leading to a nonrepresentative sample. Furthermore, women with incomplete data were excluded from the study, which might have affected the results. This could introduce bias if the excluded women had different characteristics or outcomes compared to those included in the study. The prevalence estimates for NCDs were based on hospital records, which may not accurately reflect the true prevalence in the population.

## CONCLUSIONS

The prevalence of NCDs among women of reproductive age group was higher than in other studies done in similar settings. These findings underscore the importance of early detection and intervention to prevent NCDs, particularly among women of reproductive age. Future research should focus on identifying and addressing modifiable risk factors, as well as implementing targeted interventions to promote healthy lifestyle behaviours and reduce the burden of NCDs in this population.
